# 

**Published:** 1854-12

**Authors:** 


VOL. III. NEW SERIES. No. 12.

### THE NORTH-WESTERN MEDICAL AND SURGICAL JOURNAL:

AT TWO DOLLARS PER ANNUM IN ADVANCE.

EDITED BY

N. S. DAVIS, M.D.,

PROFESSOR OF PATHOLOGY, PRACTICE OF MEDICINE AND CLINICAL MEDICINE.

AND

H. A. JOHNSON, A.M., M.D.

LECTURER ON PHYSIOLOGY AND HISTOLOGY IN RUSH MEDICAL COLLEGE.

DECEMBER, 1854.

CHICAGO:

J. F. BALLANTYNE, PUBLISHER AND PRINTER,

NEW POST-OFFICE BUILDINGS, COR. OF CLARK AND RANDOLPH-STS.,

OPPOSITE THE SHERMAN HOUSE.

VOL XI. WHOLE SERIES. NO. 12.

